# Idiopathic pulmonary hemosiderosis - a diagnostic challenge

**DOI:** 10.1186/1824-7288-40-35

**Published:** 2014-04-04

**Authors:** Ilirjana Bakalli, Luljeta Kota, Durim Sala, Ermela Celaj, Elmira Kola, Robert Lluka, Sashenka Sallabanda

**Affiliations:** 1 PICU, University Hospital Center “Mother Theresa”, Tirana, Albania; 2 Paediatric Pneumology, University Hospital Center “Mother Theresa”, Tirana, Albania

**Keywords:** Pulmonary hemosiderosis, Anemia, Children

## Abstract

Idiopathic pulmonary hemosiderosis is a rare disorder that can occur at any age and is characterized by the triad of hemoptysis, iron deficiency anemia and diffuse pulmonary infiltrates. The clinical course is exceedingly variable especially in children and a substantial proportion of this age group is undiagnosed. It is probably due to the fact that iron deficiency anemia may be the first and the only manifestation of IPH, preceding other symptoms and signs by several months and IPH is not considered as a rare cause of anemia, unless the typical triad is present. We present a case of IPH in a 13-year-old girl, treated for several months with persistent iron deficiency anemia, without response to therapy.

## Introduction

Idiopathic pulmonary hemosiderosis (IPH) is a rare and life threatening condition, found primarily in children, that causes recurrent episodes of diffuse alveolar hemorrhage. It is characterized by hemoptysis, alveolar infiltrates on chest radiograph and various degrees of anemia, seen more frequently in children than in adults [1-7]. The estimated incidence of IPH in children is 0.24–1.23 cases per million, with a mortality rate as high as 50%. Only 500 cases have been described in medical literature [3,6,8]. When no underlying cause for repeated episodes of diffuse alveolar hemorrhage is apparent, the entity is referred to as idiopathic pulmonary hemosiderosis [2]. The rarity of this disease and the variable clinical course results in many diagnostic pitfalls especially in children [9]. Late diagnosis of IPH may allow complications and the beginning of treatment at a stage when pulmonary fibrosis has already developed, with consequently poorer prognosis [2,10]. Through our presentation we want to emphasize the importance of early recognition of this syndrome.

## Case report

**Figure 1:**
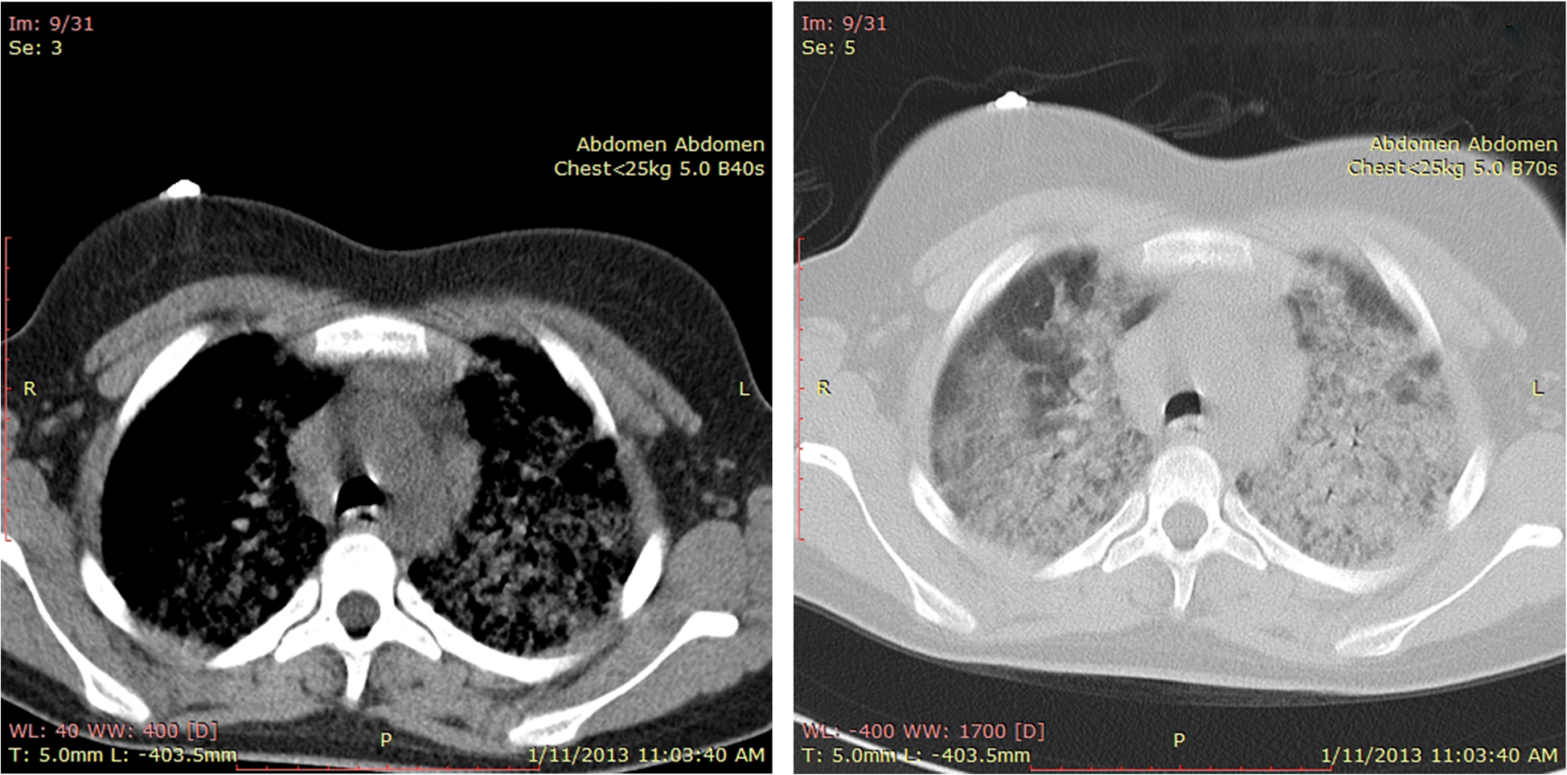
CT scan of the thorax showing diffuse alveolar interstitial infiltration.

A 13-year-old girl was admitted in hospital after an episode of faintness. At admission the girl presented very pale, with extreme tiredness and unable to stay upright, with dyspnea and polipnea, unable to maintain oxygen saturation in the air room (Sat O_2_ = 78%), with low blood pressure 80/40 mmHg. For several months the girls has been treated for iron deficiency anemia with oral administration of iron by the hematologist, but without response to therapy. A month ago, the child was again hospitalized for severe anemia with blood transfusion. Given that at the age of 3 years, the child was treated for idiopathic thrombocytopenic purpura (ITP), the first suspicion was for menorrhagia, probably due to chronic ITP, complicated with severe anemia. By the first laboratory data we observed: severe anemia with hemoglobin level of 5.4 g/dL, Red Blood Cells (RBC) - 1.9 T/L, microcytosis, hypochromia, with normal platelet number. The value of mean corpuscular hemoglobin (MCH), mean corpuscular volume (MCV), mean cell hemoglobin concentration (MCHC) and serum iron were very low. Renal and liver functions were normal. The electrocardiogram and the echocardiogram were both normal. Bone marrow biopsy shows hyperplastic erythropoiesis. No abnormality has been observed by the gynecologist. The child’s condition has deteriorated rapidly with significant respiratory failure. Chest X-ray represents bilateral alveolar infiltrates. Despite that there was no history for hemoptysis, clinical suspicion before the triade (quick anemia, respiratory failure and low levels of iron) was to pulmonary hemosiderosis. CT images (Figure 1) and the presence of hemosiderin-laden macrophages called siderophages (pathognomonic of this disease) in gastric lavage fluid confirmed the diagnosis of IPH.

Laboratory investigations revealed negative work-up for antinuclear antibodies (ANA), antineutrophil cytoplasmic antibodies (ANCA), rheumatoid factor, anti-citrullinated peptides (anti-CCP), antiglomerular basement membrane (antiGBM), antigliadin, specific cow’s milk IgE and complement. Therapy with corticosteroids was initiated with a partial and transient response. The girl presented with recurrent episodes of dyspnea and severe anemia every two-three months. Due to prolonged therapy, the girl has become progressively cushingoid. Azathioprine, the “second line” immunosuppressant recommended in IPH [2], was subsequently introduced. Now clinical situation is better controlled.

## Discussion

The triad of iron deficiency anemia, hemoptysis and diffuse infiltrates on chest - X ray, characterizes the onset of IPH. However, any of these features may be the first presenting manifestation and the clinical course is exceedingly variable especially in children [11,12]. So, from a Romanian experience of 15 children diagnosed with IPH during a 22-year period (1984–2006), the classical triad was found in only 4 cases [10]. On the other hand all patients, from the beginning had anemia and only 6 children presented with pulmonary symptoms. Even in the French RespiRare® cohort anemia and dyspnea were the most frequent clinical features at the beginning (64% and 68% respectively), while hemoptysis occurred only in 50% of the patients [1].

Young children usually swallow their sputum; as a result, hemoptysis is rare in children unless the bleeding is substantial [1,4,8]. Even in our case we didn’t find the presence of hemoptysis.

The age of presentation is bimodal, with a frequency peaks in children less than five years of age and in adolescents 11 years or older.

A substantial proportion of the first group is undiagnosed. It is probably due to not only to the lack of hemoptysis, but to the fact that iron deficiency anemia may be the first and the only manifestation of IPH, preceding other symptoms and signs by several months [3,4,13,14]. Iron deficiency anemia is the most common hematologic disorder seen in childhood and IPH is not considered as a rare cause of anemia, unless the typical triad is present [3,15].

Anemia in these cases is classical of iron deficiency, hypochromic microcytic, due to chronic loss of blood. But if in iron deficiency anemia, hematologic response to iron is usually remarkable within the first month of therapy, therapy failed during hemosyderosis [9]. As in our case, no response to therapy and need for several transfusions has been reported even in other cases [15-21]. Failure of iron therapy and exclusion of its common causes, prompt us to look at other diagnostic possibilities including IPH, even it is a rare cause.

If bleeding is not substantial, pulmonary involvement may not been found clinically at the beginning of the disease and even chest X-ray may present normal, but with the development of the disease, roentgenographic manifestations, once abnormal, never return to normal although acute massive infiltration partially resolve, making chest X-ray very useful in such cases [1]. Unfortunately, we don’t have a chest X ray of our case before the 2^nd^ hospitalization.

IPH is a severe condition with variable prognosis and has a better outcome when diagnosis is made at an early age [3,10,15]. Due to the rarity of the disease and the variability in clinical presentation, diagnosis is usually delayed. (Several months in our case) Kipper et al. noted there is a long delay (4 moths–10 years) between the starting of symptoms and the time of correct diagnosis [22]. A mean delay of 2.4 years before diagnosis was found in the Romanian experience and 30 months in the Indian experience [10,19].

The gold standard for IPH diagnosis is lung biopsy [19]. On the other hand, diagnosis of IPH can be confirmed by bronchoscopy with bronchoalveolar lavage, showing hemosiderin-laden macrophages [6,23]. In our case confirmation of the diagnosis was made by the presence of siderophages in gastric lavage fluid, which is equally diagnostic and also the simplest, reliable test in infants and young children [6].

HR-CT scan are useful for early detection of pulmonary fibrosis. Aggressive therapy is imperative to avoid pulmonary fibrosis and mortality in these patients [24].

There are no evidence-based recommendations regarding the treatment of IPH. As indicated in small case series and/or case reports, corticosteroids are the mainstay treatment of IPH [10,18]. It has been reported that corticosteroids are associated with decreased pulmonary bleeding relapses and pulmonary fibrosis [1,4,13]. Even though, there are controversial and lack clear evidence in their favor. Other studies suggest that they do not have any effect on the prognosis of this disease [11]. In the Romanian experience treatment with corticosteroids alone was not effective in 12 of 15 children [12]. Immunosuppressive therapies, mainly azathioprine and hydroxychloroquine, are mostly proposed in situations of patients with steroid-refractory disease [1,23,25]. In our case corticosteroids were partially effective. Clinical situation was better controlled with azathioprine in combination with corticosteroids.

Yoachimescu et al. also concluded that azathioprine in combination with corticosteroids might be the best therapeutic regimen, especially in preventing IPH exacerbations, as in our case [8].

According to the Indian experience, treatment with prednisolone and hydroxychloroquine were more effective in the clinical course and the survival [25].

A 2007 scientific letter, reports preliminary success in preventing pulmonary hemorrhage with the anti-oxidant N-acetylcysteine [11].

The IPH and ITP may have occurred coincidentally in this patient, but certain pathophysiological similarities between these two disorders suggests that they are related. In fact, according Mayo Clinic Internal Medicine, IPH has been described in association with ITP, autoimmune hemolytic anemia and celiac disease [26]. Even though the etiology of IPH remains unknown, there is support for an autoimmune contribution in IPH physiopathology. In the French RespiRare® cohort most of the patients had autoimmune antibodies at onset or appeared during the follow-up. (Anti-smooth-muscle antibodies SMA – 50% of the tested patients, ANA – 45% and ANCA – 40%) [1]. Work-up for autoimmune antibodies in our case resulted negative.

IPH prognosis seems to improve over time. While two decades ago the mean survival was 3 years from diagnosis, recent data show a 5-year survival in 86% of cases [3]. The significant improvement is possibly due to the long-term use of immunosuppressant therapy.

## Conclusion

IPH although a rare entity, should be considered in patients with long-term iron deficiency anemia. A chest X-ray is compulsory in every unexplained iron deficiency anemia exploration.

Pediatrician should keep in mind the possibility of pulmonary hemosiderosis, in case of very quick anemia, associated with early signs of respiratory failure.

Lack of hemoptysis does not exclude the diagnosis of pulmonary hemosiderosis.

### Consent

Written informed consent was obtained from the patient’s parent for the publication of this report and any accompanying images.

## Abbreviations

IPH: Idiopathic pulmonary hemosiderosis; ITP: Idiopathic thrombocytopenic purpura; RBC: Red Blood Cells; MCH: Mean corpuscular hemoglobin; MCV: Mean corpuscular volume; MCHC: Mean cell hemoglobin concentration; ANA: Antinuclear antibodies; ANCA: Antineutrophil cytoplasmic antibodies; anti-CCP: Anti-citrullinated peptides; antiGBM: Antiglomerular basement membrane; SMA: Anti-smooth-muscle antibodies; HR-CT scan: High resolution computed tomography scans.

## Competing interests

The authors declare that they have no competing interests.

## Authors’ contributions

IB collected the patient data and drafted the manuscript. EC, DS helped to draft the manuscript. EK helped to collect the data and draft the manuscript. IB, LK and SS participated in the diagnosis and critically revised the manuscript. All the authors approved the final version of the manuscript.
